# Predictive value of circulating coagulation related microRNAs expressions for major adverse cardiac and cerebral event risk in patients undergoing continuous ambulatory peritoneal dialysis: a cohort study

**DOI:** 10.1007/s40620-019-00626-x

**Published:** 2019-07-29

**Authors:** Yin Wang, Changxuan Liu, Wei Wei, Wenli Chen

**Affiliations:** 1 Division of Nephrology, The Central Hospital of Wuhan, Tongji Medical College, Huazhong University of Science and Technology, 26 Shengli Street, Jiang’an District, Wuhan, 430014 China; 2 Department of Respiration, The Central Hospital of Wuhan, Tongji Medical College, Huazhong University of Science and Technology, Wuhan, China

**Keywords:** Major adverse cardiac and cerebral event, End-stage renal disease, Continuous ambulatory peritoneal dialysis, MicroRNA, Survival

## Abstract

**Background:**

We aimed to investigate the correlation of coagulation related microRNAs (miRNAs) expressions with major adverse cardiac and cerebral event (MACCE) risk in patients undergoing continuous ambulatory peritoneal dialysis (CAPD).

**Methods:**

198 end-stage renal disease (ESRD) patients underwent CAPD were consecutively recruited in this study. Clinical characteristics as well as physiological and biochemical indexes were recorded. Peripheral blood was collected after enrollment to separate plasma, and 13 blood coagulation related miRNAs were detected by the real-time quantitative polymerase chain reaction. All patents were followed up for 48 months, and the last follow-up date was 2018/12/31. MACCEs occurred during the follow up were documented, and MACCE-free survival was calculated.

**Results:**

MACCE incidence at 1 year, 2 year, 3 year and 4 year was 2.5, 6.1, 9.1 and 13.1% respectively, and mean MACCE-free survival was 45.2 (95% CI 44.0–46.4) months. Kaplan–Meier curves showed that miR-30e-5p, miR-92a-3p, miR-106a-5p and miR-126-5p high expressions were associated with longer MACCE-survival, while miR-423-5p high expression correlated with shorter MACCE-free survival. Multivariate Cox’s regression analysis disclosed that miR-92a-3p, miR-126-5p and miR-652-3p independently predicted longer MACCE-free survival, while miR-423-5p independently predicted reduced MACCE-free survival in CAPD patients.

**Conclusion:**

Circulating miR-92a-3p, miR-126-5p, miR-652-3p and miR-423-5p exhibit potential to serve as novel biomarkers for MACCE risk in patients undergoing CAPD.

## Introduction

End-stage renal disease (ESRD) refers to the chronic kidney disease in the final stage, which affects over two million individuals worldwide and results in more than tenfold higher mortality rate compared to non-ESRD cases [1]. During the past decades, dialysis is a major treatment frequently used in ERSD patients to sustain life. As a stable and convenient kind of dialysis, continuous ambulatory peritoneal dialysis (CAPD) presents well performance on allowing ESRD patients to maintain renal function and improve quality of life [2–4]. Nevertheless, major adverse cardiac and cerebral event (MACCE) is a common and severe complication for ESRD patients undergoing CAPD, which leads to around 33% of hospitalizations and 41% of deaths in ESRD patients undergoing CAPD [5–7]. From the perspective of underlying pathogenesis of MACCE, it is well known that the balance between pro-coagulation and anticoagulation activities limits blood clot formation to prevent thrombus, otherwise, disruption of this equilibrium results in thrombosis and contributes to MACCE occurrence [8]. Therefore, exploration of novel biomarkers that are related to coagulation may decrease the MACCE risk and improve the survival after MACCE in ESRD patients undergoing CAPD.

MicroRNAs (miRNAs), a type of non-coding RNAs, are vital post-transcriptional modulators, which participate in a myriad of physiological functions and pathological conditions (such as endothelial homeostasis, glucolipid metabolism as well as microangiopathy), and part of them have been found to regulate several hemostatic factors, tissue-factors or anti-angiogenic factors to play essential roles in coagulation that is a critical step for thrombosis of MACCE [9–12]. Considering the crucial status of coagulation on affecting MACCE occurrence as we mentioned above, and miRNAs have regulatory effects in coagulation process, we hypothesized that the blood coagulation related miRNAs might be associated with MACCE risk or related prognosis, which might serve as markers for MACCE in CAPD patients, while related evidence is seldomly reported [12, 13]. In this study, 13 blood coagulation related miRNAs were selected referring to a recent review analysis focusing on hemostasis and coagulation pathways related miRNAs, and we aimed to investigate the correlation of these coagulation related miRNAs expressions with MACCE risk in patients undergoing long-term CAPD [14].

## Materials and methods

### Patients

From January 2012 to December 2014, 198 ESRD patients undergoing long-term (more than 3 years) CAPD in our hospital were consecutively recruited in this study. The inclusion criteria were as follows: (1) diagnosis of ESRD and underwent CAPD for at least 3 years; (2) age more than 18 years; (3) able to be regularly followed up, which was evaluated by the investigator according to the patients’ conditions; (4) life expectance more than 12 months. Patients were excluded from the study if they were suffering from or had a history of following conditions including hemodialysis, kidney transplantation or kidney surgery, cardiovascular and cerebrovascular diseases (such as coronary artery disease, myocardial infarction, cerebral ischemia, stroke, thromboangiitis obliterans and so on), percutaneous transluminal coronary angioplasty, bypass surgery or malignancies. Notably, the reason why we excluded patients with less than 3 years of CAPD was that: MACCE incidence in patients with less than 3 years of CAPD was low, and study with these patients might have low statistical power due to lack of events, moreover, a large bias would exist in study due to dialysis duration. Besides, pregnant or lactating women were also excluded. Approval of the present study was obtained from the Institutional Review Board of our hospital, and written informed consents were collected from all patients on the enrollment.

### Clinical data collection

Clinical characteristics of enrolled patients were recorded in the Case Report Form after enrollment, which mainly consisted of age, gender, body mass index (BMI), smoke, drink, and peritoneal dialysis duration. Laboratory tests were also documented, such as the value of Kt/V [which was calculated as clearance (K) multiplied by treatment time (t) and divided by the urea distribution volume (V)], hemoglobin (Hb), white blood cell (WBC), platelet, C-reactive protein (CRP), serum creatinine (Scr), serum uric acid (SUA), calcium, phosphorus, fasting blood glucose (FBG), albumin (ALB), systolic blood pressure (SBP), diastolic blood pressure (DBP), total cholesterol (TC), triglyceride (TG), low density lipoprotein cholesterol (LDL-C) and high density lipoprotein cholesterol (HDL-C).

### Blood sample collection and processing

Peripheral blood was collected from patients using ethylene diamine tetraacetic acid (EDTA) tubes, and were immediately centrifuged at 4000×*g* for 10 min to separate plasma, then the isolated plasma were placed into a − 80 °C freezer for further detection.

### MicroRNAs detection

A total of 13 blood coagulation related microRNAs were selected referring to a recent review analysis focusing on hemostasis and coagulation pathways related microRNAs [14]. And the relative expressions of 13 microRNAs in the plasma of patients were detected by the real-time quantitative polymerase chain reaction (RT-qPCR). Brief procedures were as follows: total RNA was extracted from plasma samples using QIAamp RNA Blood Mini Kit (Qiagen, Duesseldorf, Nordrhein-Westfalen, German), and then reverse transcription of RNA was performed by ReverTra Ace^®^ qPCR RT Master Mix (Toyobo, Osaka, Kansai, Japan). Subsequently, qPCR was performed by SYBR^®^ Green Realtime PCR Master Mix (Toyobo, Osaka, Kansai, Japan). U6, as an internal reference, was used to normalize the expression of miRNAs. Primers used in the RT-qPCR were listed in the Table 1.

**Table 1:** Baseline characteristics of CAPD patients

Characteristics	CAPD patients (*N* = 198)
Age (years), mean ± SD	53.0 ± 11.0
Gender (male/female), No.	128/70
BMI (kg/m^2^), mean ± SD	21.7 ± 2.6
Smoke, No. (%)	81 (40.9)
Drink, No. (%)	75 (37.9)
Peritoneal dialysis duration (months), median (IQR)	62.0 (48.0–81.0)
Kt/V, median (IQR)	1.9 (1.7–2.1)
Hb (g/L), mean ± SD	102.9 ± 16.3
WBC (X10^9^/L), mean ± SD	7.8 ± 2.2
Platelet (X10^9^/L), mean ± SD	210.2 ± 55.8
CRP (mg/L), median (IQR)	4.1 (2.4–6.8)
Scr (μmol/L), mean ± SD	937.0 ± 272.1
SUA (μmol/L), mean ± SD	415.8 ± 77.2
Calcium (mmol/L), mean ± SD	2.2 ± 0.2
Phosphorus (mmol/L), mean ± SD	1.6 ± 0.4
FBG (mmol/L), median (IQR)	5.8 (4.4–7.0)
ALB (g/L), mean ± SD	37.0 ± 5.9
SBP (mmHg), mean ± SD	142.9 ± 19.2
DBP (mmHg), mean ± SD	85.8 ± 8.8
TC (mmol/L), mean ± SD	4.7 ± 1.0
TG (mmol/L), median (IQR)	1.7 (1.0–2.4)
LDL-C (mmol/L), mean ± SD	2.8 ± 0.6
HDL-C (mmol/L), mean ± SD	1.0 ± 0.3

*CAPD* continuous ambulatory peritoneal dialysis, *SD* standard deviation, *BMI* body mass index, *IQR* interquartile range, *Kt/V* urea clearance index, *Hb* hemoglobin, *WBC* white blood cell, *CRP* C-reactive protein, *Scr* serum creatinine, *SUA* serum uric acid, *FBG* fasting blood glucose, *ALB* albumin, *SBP* systemic blood pressure, *DBP* diastolic blood pressure, *TC* total cholesterol, *TG* triglyceride, *LDL-C* low-density lipoprotein cholesterol, *HDL-C* high-density lipoprotein cholesterol

### Follow up

After initiation of study, patients were followed up every 1-3 months by outpatient clinic visits or by telephone and/or medical questionnaires. All patents were followed up for 48 months, and the last follow-up date was 2018/12/31. The MACCE occurred during the follow up were documented, which included the events of death or hospitalization caused by cardiovascular disease or cerebrovascular disease, and the events included acute coronary syndrome, stable angina pectoris requiring target vessel revascularization, transient ischemic attack and ischemic stroke. Patients were excluded from the final analysis if they lost follow up or withdrew the informed contents during follow up. MACCE-free survival was defined as the date from enrollment to the date of the occurrence of MACCE.

### Statistical analysis

Data analysis was performed by use of SPSS 24.0 software (SPSS Inc, Chicago, IL, USA), and graphs were plotted using GraphPad Prism 7.02 software (GraphPad Software Inc., San Diego, CA, USA). Data were mainly displayed as mean ± standard deviation (SD), median and interquartile range (IQR), range (min–max) or count (percentage). In detail, continuous variables were checked for normality by using the Kolmogorov–Smirnov test, and the normally distributed variables were presented as mean ± SD, the non-normal distributed variables were presented as median and IQR. MACCE-free survival was displayed by Kaplan–Meier (K–M) curve, and the difference of MACCE-free survival between two groups was determined by log-rank test. Univariate Cox’s proportional hazards regression analysis was performed to assess the factors affecting MACCE-free survival, and multivariate Cox’s proportional hazards regression analysis was applied to evaluate the independent factors predicting MACCE-free survival. All tests were 2-sided, and *P* value < 0.05 was considered significant.

## Results

### Study flow

**Fig. 1:**
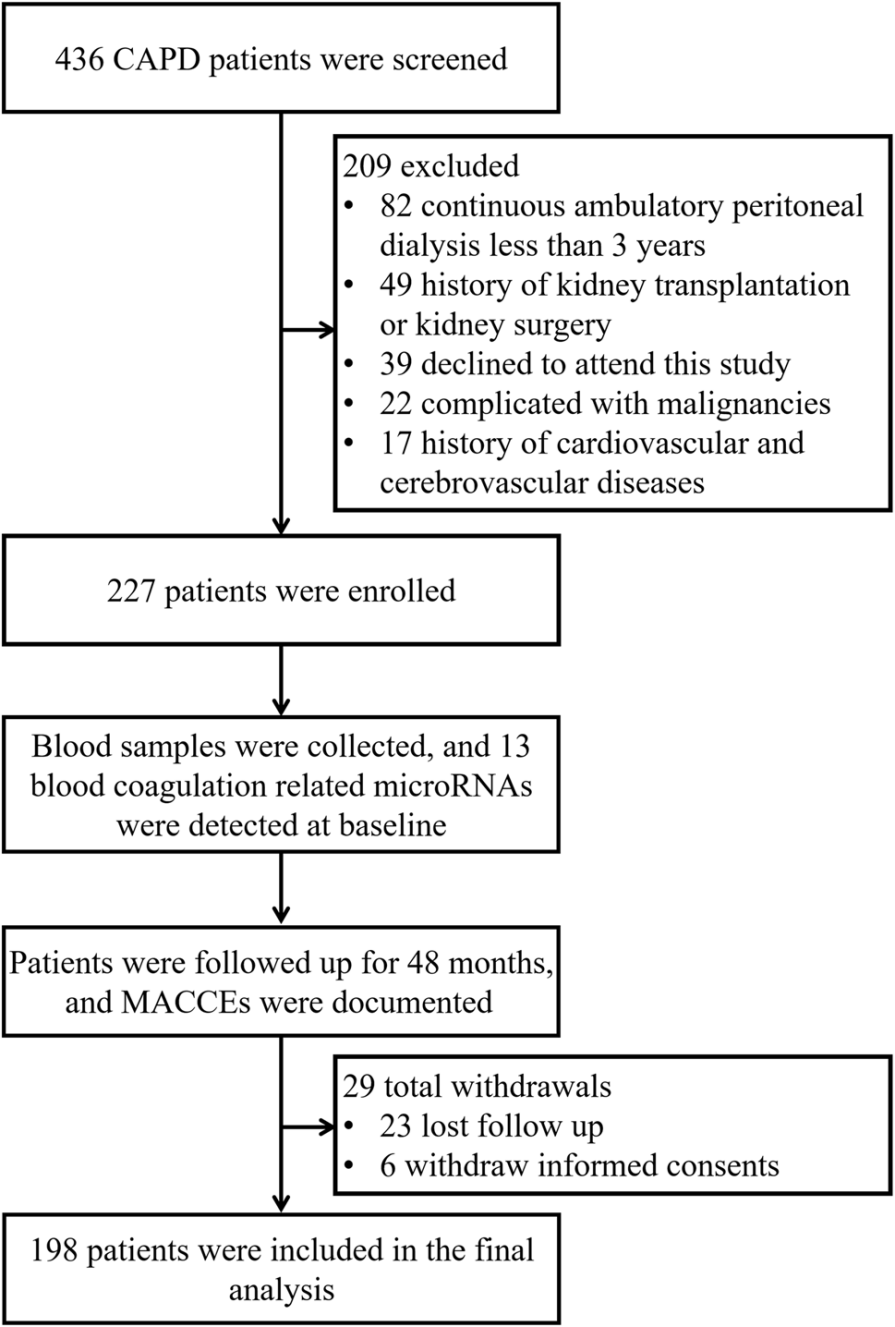
Study flow

Totally 436 CAPD patients were screened in this study, while 209 patients were excluded (including 82 patients with CAPD duration less than 3 years, 49 patients with history of kidney transplantation or kidney surgery, 39 patients declined to attend this study, 22 patients complicated with malignancies, and 17 patients with history of cardiovascular and cerebrovascular diseases) (Fig. 1). Blood samples were collected, and 13 blood coagulation related miRNAs were detected at baseline from the remaining 227 patients. These patients were followed up for 48 months, and MACCEs were documented, whereas 29 cases were withdrew (including 23 cases lost follow up and 6 cases withdraw informed consents) during the follow-up period. Finally, 198 patients were included in the final analysis.

### Baseline characteristics

Mean age of 198 CAPD patients was 53.0 ± 11.0 years (Table 1). There were 128 males and 70 females, with the mean BMI of 21.7 ± 2.6 kg/m^2^. Besides, percentage of smoke and drink were 40.9% and 37.9% respectively, and median peritoneal dialysis duration was 62.0 (48.0–81.0) months. Besides, other detailed information of baseline characteristics was displayed in Table 1.

### Blood coagulation related miRNAs expressions

13 blood coagulation related miRNAs of patients were detected, and the median values of miR-18a-5p, miR-18b-5, miR-26b-5, miR-27a-3p, miR-30e-5, miR-92a-3p, miR-106a-5p, miR-126-5p, miR-134-5p, miR-199a-3p, miR-223-3p, miR-423-5p and miR-652-3p were 2.31 (1.24–3.48), 1.16 (0.71–1.63), 1.36 (0.63–2.54), 2.25 (1.14–3.63), 0.98 (0.46–1.61), 0.93 (0.51–1.47), 1.45 (0.51–1.47), 1.00 (0.48–1.64), 1.55 (0.65–2.39), 1.18 (0.55–2.14), 1.52 (0.68–2.87), 2.11 (1.17–3.67) and 0.97 (0.41–1.54) respectively (Table 2).

**Table 2:** Blood coagulation related microRNAs relative expressions

MicroRNAs	Median	IQR
MiR-18a-5p	2.308	1.236–3.476
MiR-18b-5p	1.159	0.712–1.628
MiR-26b-5p	1.364	0.631–2.543
MiR-27a-3p	2.247	1.140–3.626
MiR-30e-5p	0.981	0.457–1.608
MiR-92a-3p	0.925	0.511–1.472
MiR-106a-5p	1.450	0.893–2.089
MiR-126-5p	0.996	0.482–1.637
MiR-134-5p	1.552	0.651–2.386
MiR-199a-3p	1.176	0.555–2.144
MiR-223-3p	1.524	0.680–2.872
MiR-423-5p	2.105	1.166–3.665
MiR-652-3p	0.972	0.412–1.539

*IQR* interquartile range

### MACCE incidence

**Fig. 2:**
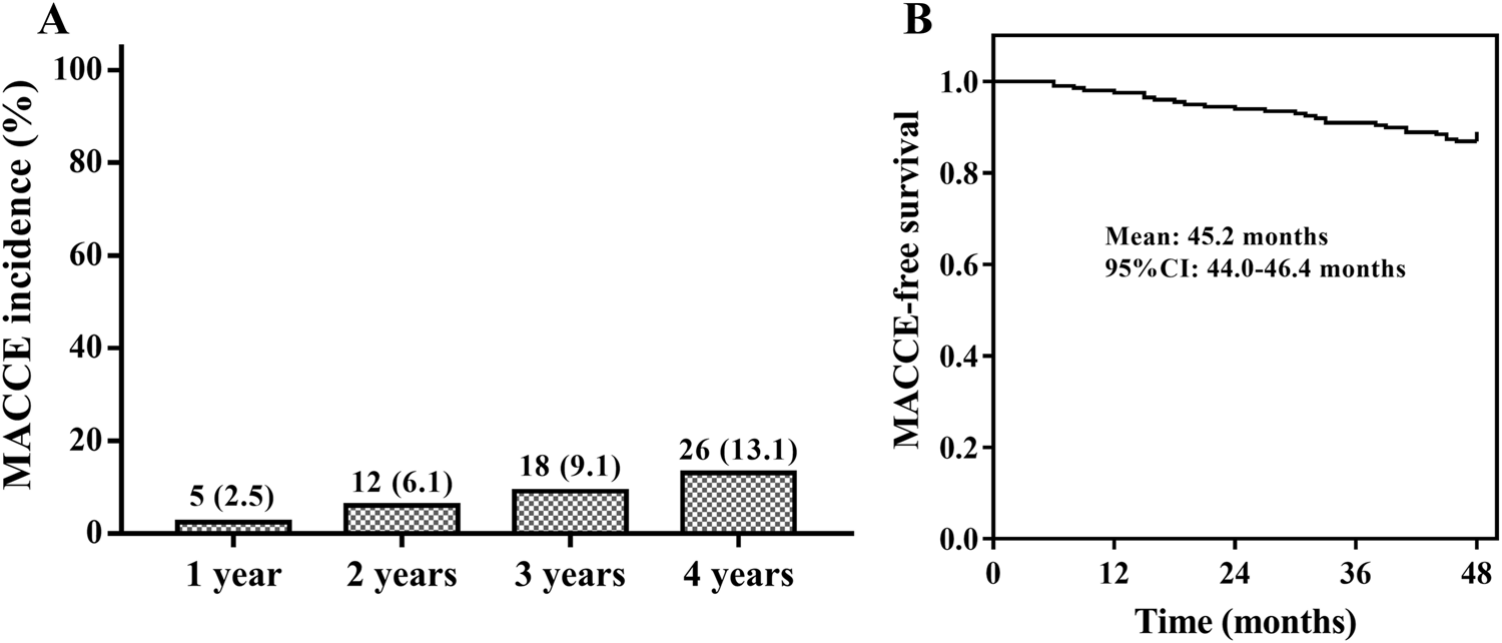
MACCE incidence and K–M curves. MACCE incidence of CAPD patients (**a**). K–M curve was performed to display the MACCE-free survival in CAPD patients (**b**). *MACCE* major adverse cardiac and cerebral event, *K–M curve* Kaplan–Meier curve, *CAPD* continuous ambulatory peritoneal dialysis

The MACCE incidence of CAPD patients at 1 year, 2 year, 3 year and 4 year was 2.5, 6.1, 9.1 and 13.1% respectively (Fig. 2a), and mean MACCE-free survival was 45.2 (95% CI 44.0–46.4) months (Fig. 2b).

### Correlation of candidate miRNAs with MACCE-free survival

**Fig. 3:**
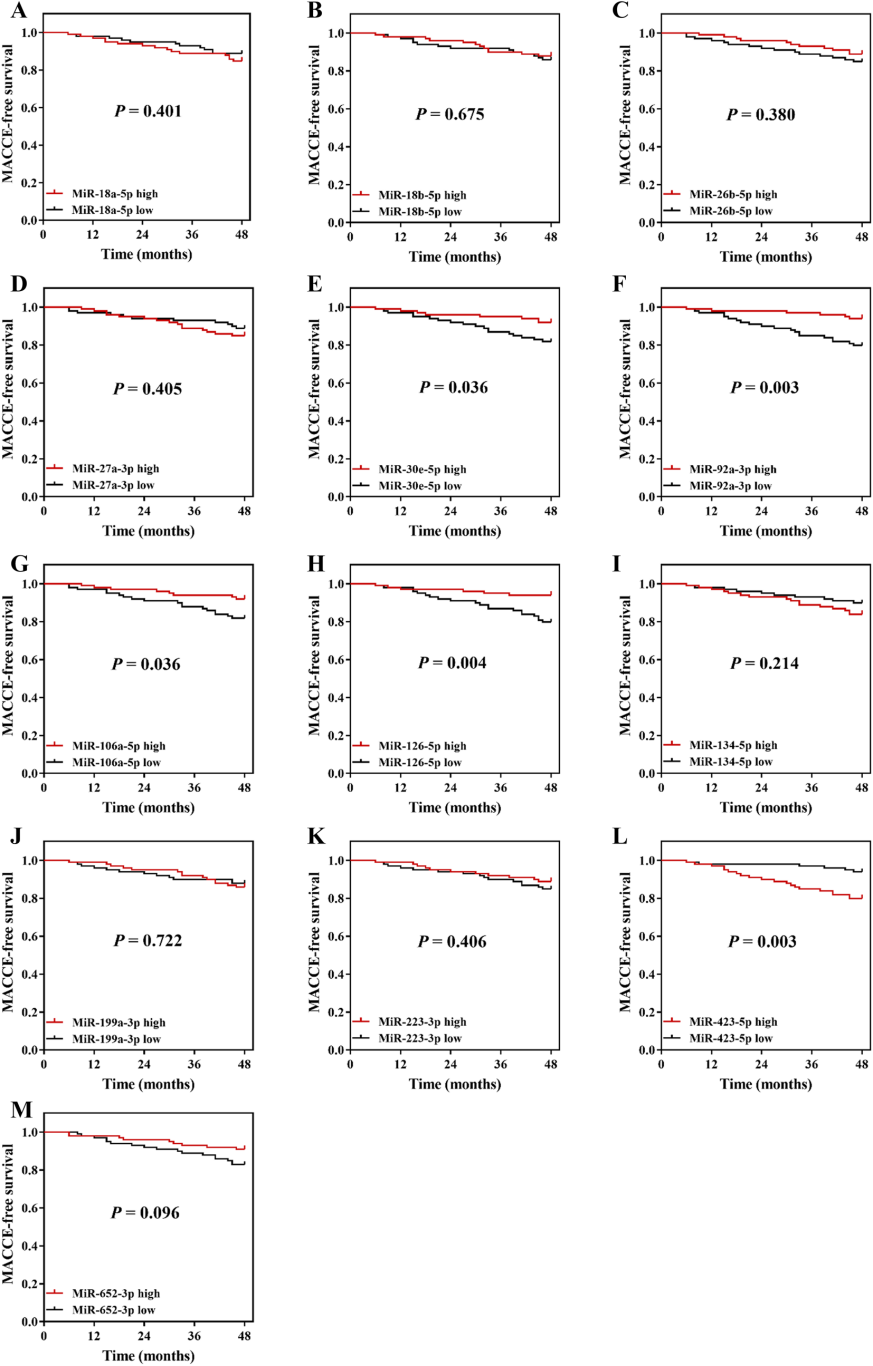
K-M curves in subgroups divided by miRNAs expressions. MACCE-free survival in miR-18a-5p high expression and miR-18a-5p low expression groups (**a**). MACCE-free survival in miR-18b-5p high expression and miR-18b-5p low expression groups (**b**). MACCE-free survival in miR-26b-5p high expression and miR-26b-5p low expression groups (**c**). MACCE-free survival in miR-27a-3p high expression and miR-27a-3p low expression groups (**d**). MACCE-free survival in miR-30e-5p high expression and miR-30e-5p low expression groups (**e**). MACCE-free survival in miR-92a-3p high expression and miR-92a-3p low expression groups (**f**). MACCE-free survival in miR-106a-5p high expression and miR-106a-5p low expression groups (**g**). MACCE-free survival in miR-126-5p high expression and miR-126-5p low expression groups (**h**). MACCE-free survival in miR-134-5p high expression and miR-134-5p low expression groups (**i**). MACCE-free survival in miR-199a-3p high expression and miR-199a-3p low expression groups (**j**). MACCE-free survival in miR-223-3p high expression and miR-223-3p low expression groups (**k**). MACCE-free survival in miR-423-5p high expression and miR-423-5p low expression groups (**l**). MACCE-free survival in miR-652-5p high expression and miR-652-5p low expression groups (**m**). K-M curve was performed to display the MACCE-free survival. Comparison between two groups was determined by log-rank test. *K-M curves* Kaplan–Meier curves, *miRNAs* microRNAs, *MACCE* major adverse cardiac and cerebral event

Patients were divided into subgroups according to the median value of these coagulation related miRNAs expressions. High expressions of miR-30e-5p (*P *= 0.036) (Fig. 3e), miR-92a-3p (*P *= 0.003) (Fig. 3f), miR-106a-5p (*P *= 0.036) (Fig. 3g) and miR-126-5p (*P *= 0.004) (Fig. 3h) were associated with longer MACCE-survival, while miR-423-5p high expression (*P *= 0.003) (Fig. 3l) was correlated with shorter MACCE-free survival in CAPD patients. No correlation of miR-18a-5p expression (*P *= 0.401) (Fig. 3a), miR-18b-5p expression (*P *= 0.675) (Fig. 3b), miR-26b-5p expression (*P *= 0.380) (Fig. 3c), miR-27a-3p expression (*P *= 0.405) (Fig. 3d), miR-134-5p (*P *= 0.214) (Fig. 1), miR-199a-3p (*P *= 0.722) (Fig. 3j), miR-223-3p (*P* = 0.406) (Fig. 3k) and miR-652-3p expression (*P *= 0.096) (Fig. 3m) with MACCE-free survival was found in CAPD patients.

### Analysis of candidate miRNAs affecting MACCE-free survival

Univariate Cox’s regression analysis displayed that miR-30e-5p (high vs. low) (P = 0.042), miR-92a-3p (high vs. low) (P = 0.006), miR-106a-5p (high vs. low) (P = 0.042) and miR-126-5p (high vs. low) (P = 0.007) were associated with better MACCE-free survival, whereas miR-423-5p (high vs. low) (P = 0.006) was correlated with worse MACCE-free survival (Table 3). Furthermore, multivariate Cox’s regression analysis disclosed that miR-92a-3p (high vs. low) (P = 0.001), miR-126-5p (high vs. low) (P = 0.009) and miR-652-3p (P = 0.042) were independent predictive factors for longer MACCE-free survival, while miR-423-5p (high vs. low) (P = 0.009) was an independent factor predicting reduced MACCE-free survival in CAPD patients (Table 4).

**Table 3:** Univariate Cox’s proportional hazard model regression analyses of candidate microRNAs affecting MACCE-free survival

Items	Univariate Cox’s regression	
	*P* value	HR (95% CI)
MiR-18a-5p (high vs. low)	0.404	1.393 (0.640–3.032)
MiR-18b-5p (high vs. low)	0.675	0.848 (0.392–1.833)
MiR-26b-5p (high vs. low)	0.383	0.707 (0.325–1.540)
MiR-27a-3p (high vs. low)	0.408	1.389 (0.638–3.025)
MiR-30e-5p (high vs. low)	0.042	0.421 (0.183–0.968)
MiR-92a-3p (high vs. low)	0.006	0.277 (0.111–0.690)
MiR-106a-5p (high vs. low)	0.042	0.421 (0.183–0.969)
MiR-126-5p (high vs. low)	0.007	0.284 (0.114–0.707)
MiR-134-5p (high vs. low)	0.219	1.642 (0.745–3.618)
MiR-199a-3p (high vs. low)	0.723	1.150 (0.532–2.486)
MiR-223-3p (high vs. low)	0.409	0.720 (0.331–1.569)
MiR-423-5p (high vs. low)	0.006	3.625 (1.455–9.028)
MiR-652-3p (high vs. low)	0.103	0.511 (0.228–1.146)

*HR* hazard ratio, *CI* confidence interval

*P* value < 0.05 was considered significant. MACCE: major adverse cardiac and cerebral event; All microRNAs were divided by median values as high and low expressions

**Table 4:** Multivariate Cox’s proportional hazard model regression analyses of candidate microRNAs affecting MACCE-free survival

Items	Multivariate Cox’s regression	
	*P* value	HR (95% CI)
MiR-18a-5p (high vs. low)	0.434	1.400 (0.603–3.250)
MiR-18b-5p (high vs. low)	0.921	1.043 (0.454–2.398)
MiR-26b-5p (high vs. low)	0.928	1.041 (0.437–2.481)
MiR-27a-3p (high vs. low)	0.465	1.369 (0.590–3.174)
MiR-30e-5p (high vs. low)	0.105	0.467 (0.186–1.172)
MiR-92a-3p (high vs. low)	0.001	0.190 (0.069–0.524)
MiR-106a-5p (high vs. low)	0.217	0.555 (0.218–1.412)
MiR-126-5p (high vs. low)	0.009	0.278 (0.107–0.725)
MiR-134-5p (high vs. low)	0.112	1.982 (0.852–4.612)
MiR-199a-3p (high vs. low)	0.856	0.926 (0.402–2.134)
MiR-223-3p (high vs. low)	0.255	0.617 (0.268–1.418)
MiR-423-5p (high vs. low)	0.009	3.823 (1.408–10.382)
MiR-652-3p (high vs. low)	0.042	0.412 (0.176–0.969)

*HR* hazard ratio, *CI* confidence interval

*P* value < 0.05 was considered significant. MACCE: major adverse cardiac and cerebral event; all microRNAs were divided by median values as high and low expressions

## Discussion

In this study, we observed that: (1) MACCE incidence at 1 year, 2 year, 3 year and 4 year was 2.5, 6.1, 9.1 and 13.1% respectively in CAPD patients, and mean MACCE-free survival was 45.2 (95% CI 44.0–46.4) months; (2) circulating miR-92a-3p expression, miR-126-5p expression and miR-652-3p expression were independent predictive factors for longer MACCE-free survival, while miR-423-5p expression independently predicted reduced MACCE-free survival in CAPD patients.

Some previous studies have investigated the prognosis related to MACCE in dialysis patients [15, 16]. For instance, a study enrolling 65 hemodialysis patients discovers a 2-year MACCE incidence of 32.3%, and another study enrolling 108 hemodialysis patients shows a median cardiovascular disease-free survival of 7.63 years (95% CI 7.06–8.21 years) [15, 16]. Considering the sample sizes in these previous studies (65 and 108) are relatively small and their enrolled patients were restricted on hemodialysis patients, further study with larger sample size concerning CAPD patients is needed. In our study, we enrolled 198 ESRD patients undergoing CAPD (a relatively larger sample size), and we found that MACCE incidence at 1 year, 2 year, 3 year and 4 year was 2.5, 6.1, 9.1 and 13.1% respectively, and the mean MACCE-free survival was 45.2 (95% CI 44.0–46.4) months in CAPD patients, these results might be different compared to those previous studies, and the reasons might be: (1) MACCE incidence of our study was lower than that in one previous study, which might be due to the lower age as well as shorter dialysis duration of enrolled patients in our study [15]; (2) MACCE-free survival in our study was numerically shorter than another previous study, which might be due to the decreased follow-up duration of our study compared to the previous study [16].

Multiple events of MACCE (such as venous thromboembolism, coronary artery disease, and ischemic stroke) are mainly caused by thrombosis that is a consequence of the disrupted equilibrium between pro-coagulation and anti-coagulation [8, 17, 18]. In order to decrease the MACCE risk and the related mortality in CAPD patients, mounting studies are performed and they reveal that miRNAs have been identified as the regulators in various physiological functions and pathological conditions, including coagulation process [8, 14, 17–19]. According to previous data, miRNAs might participate in coagulation mainly through the following mechanisms: (1) directly targeting hemostatic factors (such as fibronectin (FN1) and fibrinogen β chain (FGB)); (2) mediating expression of tissue-factor, which is a critical initiating factor of coagulation; (3) presenting impact on the anti-angiogenic factor, thrombospondin-1 (encoded by THBS1) [8]. Based on these understandings, these blood coagulation related miRNAs might underly the pathology of MACCE and eventually affect MACCE occurrence or be associated with the MACCE related prognosis. Hence, in the current study, we selected 13 coagulation related miRNAs referring to a recent review analysis, and we assessed the association of these candidate miRNAs with MACCE-free survival in ESRD patients undergoing CAPD. We found that high expressions of miR-30e-5p, miR-92a-3p, miR-106a-5p and miR-126-5p were associated with longer MACCE-survival, while miR-423-5p high expression was correlated with shorter MACCE-free survival in CAPD patients, and these results might be due to the regulation of these miRNAs on hemostatic factor, tissue-factors and anti-angiogenic factors [8].

miR-92a-3p, which belongs to the miR-17-92 cluster, is identified as a blood coagulation related miRNA that associated with disease risk of some cardiovascular diseases [11, 14, 20, 21]. For instance, a study shows that miR-92a-3p expression were downregulated in blood samples of congestive heart failure patients compared to healthy controls [21]. Also, miR-92a-3p high expression has been found correlated with decreased atherosclerosis and heart failure risk [11, 21]. These studies indicate that miR-92a-3p may play a favorable role in preventing cardiovascular diseases, while its predictive value for prognosis related to MACCE is rarely reported. In our study, we found that miR-92a-3p high expression was an independent predictive factor for longer MACCE-free survival, and the reason might be that: miR-92a-3p raised regional athero-susceptibility through targeting hemostatic factors, thereby contributed to protection in arterial endothelium, which reduced the risk of disorder in cardio-cerebral-vascular system and eventually prolonged the MACCE-risk in CAPD patients [11, 22].

miR-126, as the most abundantly expressed miRNA in endothelial cells, plays a key role in vascular development, integrity and response to hemodynamic stress, moreover, its dysregulation is related to MACCE occurrence [23, 24]. For instance, miR-126 high expression is associated with reduced CAD risk as well as lower atherosclerosis risk [11, 25]. For correlation of miR-126 expression with MACCE-free survival, only a study discloses that miR-126 high expression predicts longer MACCE-free survival in coronary artery disease patients [18]. These data reveal that miR-126 expression is negatively correlated with MACCE risk but positively associated with MACCE-free survival. In line with these studies, our study observed that miR-126-5p high expression was an independent predictive factor for longer MACCE-free survival, which might due to: miR-126-5p promoted endothelial proliferation through repressing Notch1 inhibitor delta-like 1 homolog (Dlk1) and facilitated VEGF signaling via suppressing SPRED1 and PIK3R2/p85-β, thereby decreased endothelial dysfunction and repressed coagulation to prevent formation of atherosclerotic lesion, thus reduced MACCE risk and led to better MACCE-free survival in CAPD patients [23, 26].

miR-652-3p has been recently identified as a cancer related gene, while the role of miR-652 in CAPD patients is rarely reported [27, 28]. For cardiovascular and cerebrovascular diseases, an experiment shows that miR-652-3p downregulates MTP18 and attenuates mitochondrial fission, cardiomyocyte apoptosis, and MI in vitro and in vivo [27]. And a clinical practice displays that miR-652 low expression is associated with increased atherosclerotic disease risk and worse cardiovascular-related outcome in patients with heart failure [28]. These studies reveal that miR-652 may function as a protective role in cardiovascular and cerebrovascular diseases. In our study, we found that miR-652-3p high expression was an independent factor predicting better MACCE-free survival in patients undergoing CAPD, and this result might be due to its inhibition on cardiomyocyte apoptosis [27].

miR-423-5p locates within the intron of nuclear speckle splicing regulatory protein 1 (NSRP1), is also a blood coagulation related microRNA. Despite the detailed biological function of miR-423-5p is largely unclear, several studies have disclosed the role of miR-423-5p in cardiovascular and cerebrovascular diseases [12, 29, 30]. For example, a study displays that miR-423-5p high expression is correlated with increased venous thromboembolism [12]. And some other studies show that miR-423-5p expression is upregulated in heart failure patients and correlates with severer disease status of cardiac ischemia [30–32]. These data display that miR-423-5p may serve as biomarker for higher risk and advanced progression of MACCE, indicating miR-423-5p might have unfavorable influences in MACCE. In line with these data, our study found that MiR-423-5p high expression was an independent factor predicting reduced MACCE-free survival in CAPD patients, and the reason might be that miR-423-5p was implicated in myocardial signaling networks and it triggered cardiac cell apoptosis as well as accelerated coagulation, thus it increased endothelial dysfunction and aggravated disease status, which further led to shorter MACCE-free survival in CAPD patients [29].

Some limitations still existed in our study: (1) this was a single center study, which might lack wide representativeness; (2) follow-up time of 48 months might be relatively short, and long-term effect of blood coagulation related miRNAs in CAPD patients was not explored; (3) there was an approximately 10% lost follow-up rate in our study, which might result from that a proportion of patients underwent followed treatment in other nearer hospital due to change of permanent location; (4) only the expressions of blood coagulation related miRNAs at baseline were measured, whereas the change of these miRNAs during follow-up was not detected, thus the effect of these miRNAs on monitoring disease progression in CAPD patients was not evaluated.

In conclusion, circulating miR-92a-3p, miR-126-5p, miR-652-3p and miR-423-5p exhibit potential to serve as novel biomarkers for MACCE risk in patients undergoing CAPD. These data would provide new insight into the application of coagulation related miRNAs as markers for MACCE in CAPD patients.
